# An enhanced isothermal amplification assay for viral detection

**DOI:** 10.1038/s41467-020-19258-y

**Published:** 2020-11-20

**Authors:** Jason Qian, Sarah A. Boswell, Christopher Chidley, Zhi-xiang Lu, Mary E. Pettit, Benjamin L. Gaudio, Jesse M. Fajnzylber, Ryan T. Ingram, Rebecca H. Ward, Jonathan Z. Li, Michael Springer

**Affiliations:** 1 Department of Systems Biology, Harvard Medical School, Boston, MA 02115 USA; 2 Laboratory of Systems Pharmacology, Harvard Medical School, Boston, MA 02115 USA; 3 Biological and Biomedical Sciences Program, Harvard Medical School, Boston, MA 02115 USA; 4 Brigham and Women’s Hospital, Harvard Medical School, Boston, MA 02115 USA; 5 Massachusetts Consortium on Pathogen Readiness, Boston, MA 02115 USA

**Keywords:** PCR-based techniques, Assay systems

## Abstract

Rapid, inexpensive, robust diagnostics are essential to control the spread of infectious diseases. Current state of the art diagnostics are highly sensitive and specific, but slow, and require expensive equipment. Here we report the development of a molecular diagnostic test for SARS-CoV-2 based on an enhanced recombinase polymerase amplification (eRPA) reaction. eRPA has a detection limit on patient samples down to 5 viral copies, requires minimal instrumentation, and is highly scalable and inexpensive. eRPA does not cross-react with other common coronaviruses, does not require RNA purification, and takes ~45 min from sample collection to results. eRPA represents a first step toward at-home SARS-CoV-2 detection and can be adapted to future viruses within days of genomic sequence availability.

## Abstract (web summary)

Current state-of-the-art diagnostics for infectious diseases are sensitive but require extensive equipment. Here the authors develop an enhanced recombinase polymerase amplification reaction for SARS-CoV-2 that allows for inexpensive and rapid testing with minimal equipment.

## Introduction

SARS-CoV-2 has rapidly spread around the world with serious consequences for human life and the global economy^1^. In many countries, efforts to contain the virus have been hampered by a lack of adequate testing^2^. Rapid, inexpensive, and sensitive testing is essential for contact tracing and isolation strategies to be effective^3^. While numerous different tests exist, the overwhelming global need for testing has led to limitations in both the supplies of reagents, e.g., swabs and purification kits, and instrumentation, e.g., quantitative polymerase chain reaction (qPCR) or ID NOW machines. In most cases, overcoming these limitations would require scaling of supply lines by several orders of magnitude over current production capacities. Therefore, in an effort to avoid overrun health care systems and high death tolls, many countries have resorted to costly lockdowns.

The ability to reopen economies safely depends crucially on the testing capacity available. Efforts to increase testing capacity have included testing from saliva^4^, using nonstandard storage media or dry swabs^5^, and eliminating the normal RNA purification step from the standard RT-qPCR tests^6,7^. Strategies such as pooling samples followed by detection using traditional or high throughput sequencing approaches have also been proposed as a way to allow significantly more testing at a highly reduced cost^8,9^. In general, such strategies force a trade-off between throughput and sensitivity.

Isothermal amplification technologies have long held promise to offer highly sensitive detection at high throughput, and to allow for widely distributed testing including at-home/point-of-need (PON) tests^10,11^. However, isothermal amplification is plagued by nonspecific amplification events that require secondary amplification and detection steps. These steps add extra complexity to the reactions, removing many of the benefits of the isothermal amplification approach. Many ongoing efforts aim to circumvent these problems for SARS-CoV-2 detection. Most of the approaches developed so far still require an extraction step and/or two amplification steps to achieve high specificity, or have low sensitivities that give poor concordance with the gold standard RT-qPCR test^11^.

We set out to determine the underlying reasons for the poor performance of isothermal amplification technologies in viral detection applications. We selected reverse transcription-recombinase polymerase amplification (RT-RPA) as the most promising current technology. RT-RPA is an isothermal amplification method in which the double stranded DNA denaturation and strand invasion that is typically achieved by heat cycling in PCR is instead accomplished by a cocktail of recombinase enzymes, single-stranded binding proteins, and DNA polymerases^12^. RPA has potential advantages over other isothermal amplification technologies such as loop-mediated isothermal amplification (LAMP)^13^ as it can be performed near ambient temperature (37–42 °C) and is more rapid. While several creative applications of LAMP technologies to diagnose COVID-19, the disease caused by SARS-CoV-2, have recently been developed and show promise^14–18^, RT-RPA has been less explored.

Here, we present a method to screen for efficient RPA primers and show that proper RT enzyme selection with the addition of RNase H to a standard RPA reaction enhances detection of viral RNA targets. We show that the enhanced RPA (eRPA) reaction allows for specific detection of SARS-CoV-2 N and S gene down to five molecules per reaction. We demonstrate how eRPA can be used to detect SARS-CoV-2 on unextracted samples from saliva or swab transport media. eRPA is validated on clinical samples and gives concordant results with RT-qPCR in all samples above five molecules per reaction. These important improvements for RPA represent a step toward at-home SARS-CoV-2 detection using isothermal amplification.

## Results

### Reverse transcriptase choice can greatly affect recombinase polymerase amplification efficiency

**Fig. 1:**
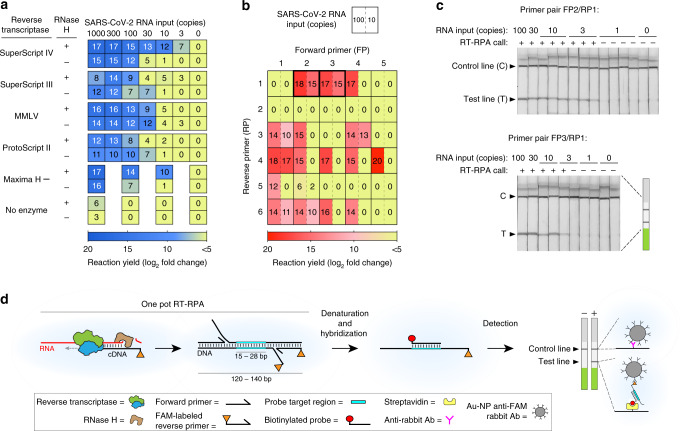
Development of eRPA: an enhanced RT-RPA based assay for detection of SARS-CoV-2. **a** Screen for reverse transcriptase (RT) enzyme and effect of RNase H. SARS-CoV-2 RNA was amplified by RT-recombinase polymerase amplification (RT-RPA) using five different RTs with or without RNase H addition and the yield of each reaction was determined by quantitative PCR (qPCR). At least two biological and two technical replicates were used for each data point; numbers in each square represent mean log_2_ fold amplification. Samples labeled as zero yielded only non-specific amplification products. **b** Primer optimization screen. SARS-CoV-2 RNA was amplified by RT-RPA using forward and reverse primers specific to the S gene. The yield of each reaction was determined by qPCR using the same primer pair as for the RT-RPA reaction. Data represent mean log_2_ fold amplification from two technical replicates for each RNA input. **c** Lateral flow strip readout of RT-RPA reactions of SARS-CoV-2 RNA using primer pairs FP2/FAM-labeled RP1 and FP3/FAM-labeled RP1. All lateral flow strips contain a control (C) and test (T) band. **d** Schematic of eRPA. Viral RNA is first copied to cDNA by RT, then degraded by RNase H. The cDNA product is amplified by RPA using a forward and a FAM-labeled reverse pair of primers specific to the target sequence. The amplified material is then denatured and hybridized to a biotinylated probe. Dual FAM-labeled and biotin-labeled products are detected on lateral flow strips. Source data are available in the Source Data file.

We designed RPA primers to both the SARS-CoV-2 N gene and S gene (Supplementary Fig. 1a and Supplementary Table 1) and quantified the amplification of a RT-RPA assay with ProtoScript II reverse transcriptase by qPCR (Supplementary Fig. 1b). The detection limit of this standard assay was poor, requiring between 100 and 300 RNA molecules for reliable detection (Fig. 1a and Supplementary Fig. 1c, bottom panel). Some studies have used longer reaction times to partially counteract the poor yield of RT-RPA^19^, but we set out to determine whether alternative approaches were possible.

We reasoned that the poor performance of RT-RPA could either be due to a specific inhibitor of the RPA reaction from the RT (reverse transcription) reaction or to nonspecific primer oligomerization products that could dominate the amplification reaction before the RT reaction occurs (Supplementary Data 1). These possibilities are not mutually exclusive. As the RPA reaction is both fast and sensitive when DNA is used as an input^12,20^, we further hypothesized that the product of the RT reaction, i.e., the RNA:DNA hybrid duplex, might inhibit the RPA reaction. We explored methods to circumvent both of these possible problems. To address the problem of kinetic interference by non-specific oligomerization, we screened multiple reverse transcriptases; and to attempt to remove interference from RNA:DNA hybrids, we introduced RNase H, which selectively degrades the RNA strand in these hybrids. Our tests showed that both RT enzyme choice and RNase H addition affected the sensitivity of the RT-RPA reaction, suggesting that both of our hypothesized mechanisms affect RT-RPA efficiency (Fig. 1a and Supplementary Fig. 1c, d). The best combination we identified was SuperScript IV reverse transcriptase with RNase H. The magnitude of the effect of the addition of RNase H was correlated with the intrinsic RNase H activity of the RT enzyme. Both SuperScript IV and Maxima H Minus reverse transcriptases are engineered to have minimal RNase H activity in order to improve their processivity, robustness, and synthesis rate^21^, and we saw the largest effect of RNase H addition in RT-RPA reactions using these enzymes.

### Reducing non-specific primer reactions increases RT-RPA yield

In addition to the performance issues addressed above, nonspecific amplification reactions of primer dimers can greatly inhibit the ability of RPA to amplify the sequence of interest^10^. To determine whether primer choice affects the importance of these nonspecific reactions, we designed forward and reverse primers to both the SARS-CoV-2 N gene and S gene (Supplementary Fig. 1a). Our primer designs avoided regions with strong homology to other coronaviruses including MERS and SARS-CoV, as well as HCoV-229E, HCoV-HKU1, HCoV-NL63, HCoV-OC43, which cause respiratory illnesses such as the common cold. We also avoided regions that have high variability across sequenced SARS-CoV-2 strains (Supplementary Data 2). Primer pairs were screened by performing qPCR on diluted RT-RPA products so that both specific and nonspecific reaction yield could be determined, using a modification of a method we previously developed (Fig. 1b)^20^. Many primer pairs gave high levels of amplification at 100 molecules of input RNA, but only a small fraction of those yielded significant amplification products at ten molecules of input RNA (Fig. 1b). We selected primer pairs that gave a high yield of the desired target sequence while minimizing the amount of nonspecific amplicons for further assay development.

### An optimized RT-RPA reaction allows for simple detection

Our optimized RT-RPA assay’s product can be hybridized and detected with a commercial lateral flow assay (LFA) without further amplification. LFAs allow accurate read-out by eye by minimally trained personnel, and even opens up the possibility of home-based testing^22^. We chose to use Milenia Biotec HybriDetect lateral flow test strips that contain a streptavidin band, an anti-Ig band, and carry gold nanoparticle-labeled anti-FAM antibodies for visualization. Based on the results shown in Fig. 1b, we selected two primer pairs that amplify part of the S gene, added a FAM label to the reverse primer, and hybridized the product amplicon to a biotinylated capture probe. Consistent with expectations from qPCR, both primer pairs reproducibly yielded bands with ten input molecules, and one gave consistent bands with three input molecules (Fig. 1c). To allow for increased sample input without sacrificing RPA amplification efficiency, we modified the manufacturer’s RPA recipe by using more concentrated reagents (see “Methods” section). Altogether, our enhanced recombinase polymerase amplification assay (eRPA) has a detection limit several orders of magnitude better than the manufacturer’s RT-RPA assay using ProtoScript II (Fig. 1d and Supplementary Fig. 1c).

### eRPA is a sensitive, specific, rapid test for SARS-CoV-2

**Fig. 2:**
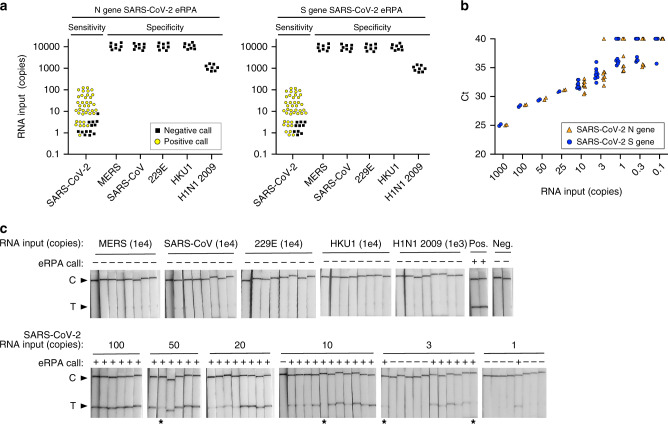
Sensitivity and specificity of RNA detection. **a** Summary of eRPA test results for detection of RNA from SARS-CoV-2 or from other viruses. Synthetic full genome SARS-CoV-2 RNA was amplified by eRPA using primers targeting the N or S gene and reactions were read out by lateral flow strip. The specificity of eRPA was tested against either in vitro transcribed (IVT) RNA of the related viruses MERS and SARS-CoV, or IVT RNA of the common cold coronaviruses HCoV-HKU1 and HCoV-229E, or viral genomic RNA extracted from 2009 H1N1 Influenza. Data points represent positive (yellow circles) or negative (black squares) eRPA tests for each sample tested and are staggered on both axes for visualization. **b** Quantification of the synthetic full genome SARS-CoV-2 RNA used as input in the eRPA assay by RT-qPCR. Data are Ct values determined using a one-step commercial RT-qPCR assay using primers targeting either the N or S gene of SARS-CoV-2. Data points at Ct = 40 represent non-specific or no amplification. N gene (orange triangles) and S gene (blue circles) data are offset on the *x*-axis for visualization purposes. **c** Lateral flow strip readouts for all N gene data shown in **a**. Individual strips are labeled with the test call made within 20 min of detection (positive (+) or negative (−)). The positive (Pos.) eRPA control is 1000 copies of synthetic full genome SARS-CoV-2 RNA and the negative (Neg.) eRPA control is a water-only input. Images taken for the purpose of display were allowed to dry which reduced the intensity of some weak bands (labeled with asterisks). Source data are available in the Source Data file.

We designed sensitivity and specificity tests of eRPA assays targeting SARS-CoV-2 N and S genes (Fig. 2 and Supplementary Fig. 2). These tests were conducted by two independent groups, each of whom randomized the RNA input in a 96-well plate in a checkerboard pattern, then handed the blinded plate to the other group for testing by eRPA (Supplementary Fig. 2a). For each gene, 52 positive samples were included with a concentration ranging from 100 molecules to 1 molecule of total RNA input (Fig. 2a, c and Supplementary Fig. 2b, c). The titer of the RNA dilutions was confirmed by RT-qPCR (Fig. 2b and Supplementary Fig. 2d–f). Strips were scored at ~20 min as this decreases the variability in band intensity that can be observed at low molecule input (Fig. 2c and Supplementary Fig. 2b). At or above ten molecules of RNA input, 87 of 88 N gene samples and 88 of 88 S gene samples were accurately identified as SARS-CoV-2 positive (Supplementary Fig. 2c). Significant detection was achieved even as low as 3 (13 of 24 tests) or 1 (5 of 16 tests) molecules of RNA input. Critically, our assay is also highly specific, showing no cross-reactivity (0 of 80 tests) with 10,000 copies of RNA from other coronaviruses, i.e., MERS, SARS-CoV, CoV-HKU1, or CoV-229E. It also showed no cross-reactivity with the 2009 H1N1 Influenza virus, a respiratory virus with similar initial clinical presentation (Fig. 2a, c and Supplementary Fig. 2b). For SARS-CoV and MERS, which have the highest target sequence identity with SARS-CoV-2 (91 and 66%, respectively), cross-reactivity is dependent on probe choice; we observed cross-reactivity with MERS and SARS-CoV when a longer biotin-probe was used for detection (Supplementary Fig. 2g).

**Fig. 3:**
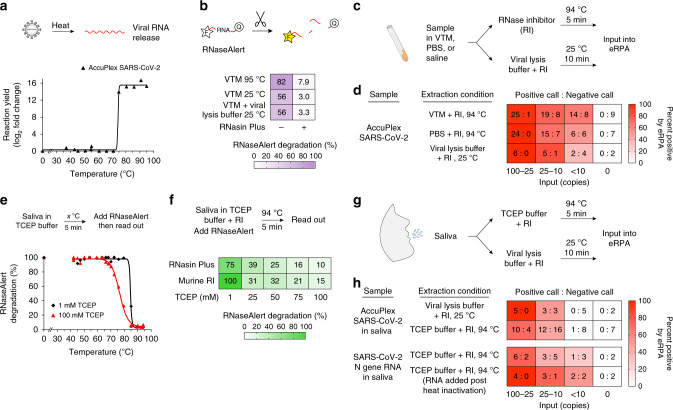
Lysis and detection of SARS-CoV-2 N gene from contrived samples. **a** Viral particle temperature lysis determination. AccuPlex packaged SARS-CoV-2 virus was diluted into TCEP buffer and heated for 5 min at the given temperature (see “Methods” section). Released RNA was amplified by eRPA and product formation was quantified by qPCR. **b** Detection of RNase activity of VTM. RNaseAlert was added to viral transport media (VTM) with or without the addition of RNasin Plus before heating for 5 min at 94 °C or added to a 1:1 VTM and viral lysis buffer mix and incubating for 10 min at 25 °C. Data represent the average of four technical replicates and were determined by normalizing the fluorescence intensity 10 min after the heating step to a fully degraded control. **c** Schematic of sample processing of patient samples in VTM for input into eRPA. **d** Heatmap displaying eRPA test calls for detection of AccuPlex packaged SARS-CoV-2 lysed with conditions displayed in **c**. AccuPlex packaged SARS-CoV-2 virus was mixed 1:1 with VTM, PBS, or viral lysis buffer and incubated as shown. All samples included RNasin Plus. Values represent the number of positive test calls: number of negative test calls for each condition. **e** Inactivation of RNase activity in saliva by TCEP and heat. Saliva was first mixed 1:1 with a buffer containing 1 mM (black diamonds) or 100 mM (red triangles) TCEP and heated at the indicated temperature for 5 min. After cooling, RNaseAlert was added and degradation was assessed as in **b**. **f** The combined activities of an RNase inhibitor and TCEP protect RNA from degradation in saliva. RNaseAlert was added to saliva diluted 1:1 with TCEP buffer containing an RNase inhibitor and treated as shown. RNAseAlert degradation was assessed an in **b**. See additional data in Supplementary Fig. 3g. **g** Schematic of sample processing of patient saliva samples for input into eRPA. **h** Heatmap displaying eRPA test calls for detection of SARS-CoV-2 RNA or AccuPlex packaged virus from saliva treated as displayed in **g**. AccuPlex packaged SARS-CoV-2 virus or SARS-CoV-2 N gene IVT RNA were added to saliva and extracted as shown. Values represent the number of positive test calls: number of negative test calls for each condition. Each experiment was repeated three times with similar results. Source data are available in the Source Data file.

We developed an RNA extraction free lysis approach as RNA extraction from clinical samples has become a limiting factor as the global need for SARS-CoV-2 tests has increased. RNA extraction kits are currently hard to obtain, the process of extraction depends on skilled workers, and often involves equipment such as centrifuges. Additionally, extraction free methods would be required for an at-home diagnostic kit. Heat-based lysis has shown promise as a way to rapidly lyse and inactivate viruses for use in diagnostic assays^23,24^. To test whether heat-based sample lysis made viral RNA accessible for eRPA, we initially used packaged reference viral particles, the AccuPlex SARS-CoV-2 verification panel (Seracare). We determined the relationship between temperature and viral lysis by heating for 5 min followed by RT-RPA then qPCR for quantification. The replication-deficient virus in the AccuPlex panel is lysed at ~75 °C, a temperature that is likely similar to the temperature required to lyse wild-type SARS-CoV-2 (Fig. 3a)^25^.

RNase inhibitors prevent RNA degradation from nasopharyngeal (NP) swabs suspended in viral transport media (VTM), the standard for clinical samples. Our initial experiments using AccuPlex samples or in vitro transcribed (IVT) RNA in VTM yielded poor signal intensities by eRPA. Using RNaseAlert to measure RNase activity, we were surprised to find significant RNase activity in VTM (Fig. 3b). In an attempt to address this we tested TCEP, which has been used to inactivate RNases from saliva and urine^26^. Unfortunately, TCEP and heat treatment of samples with VTM led to gelation, likely due to the presence of gelatin and bovine serum albumin in VTM (Supplementary Fig. 3a). As an alternative we tested RNasin Plus, a thermostable RNase inhibitor, which significantly protected RNaseAlert from degradation during heat-based lysis in VTM. For future compatibility with PON testing, we also tested a room temperature viral lysis buffer (Intact Genomic FastAmp® Viral and Cell Solution for Covid-19 Testing) and found that RNaseAlert was protected from degradation in the presence of RNasin Plus (Fig. 3b and Supplementary Fig. 3b, c).

To confirm that this protocol is effective for patient samples, we tested heat-based lysis of NP-swabs in VTM in the absence and presence of RNasin Plus. We found the addition of RNasin Plus increased RNA yield by ~10-fold and significantly improved the sensitivity of eRPA (Supplementary Fig. 3b, c). We also measured the sensitivity of eRPA for AccuPlex viral particles diluted into VTM, PBS, or viral lysis buffer. Sensitivity in these simulated samples, which should more closely reflect what would be achieved from standard samples, was reduced by about 5-fold in comparison to RNA samples in water (Figs. 2 and 3d). Most patients during the initial active phase of infection deliver NP swabs with virus concentrations of >10^4^ per mL, well within our detection limit^27–30^.

### Adaptation of eRPA to detect virus in saliva

Given the bottleneck in NP swabs, there has been growing interest in testing saliva instead^31^. Saliva is a challenging fluid due to the presence of mucins and RNases^32,33^ which can degrade RNA and clog pipettes, leading to a high rate of failed experiments or false negatives. Nevertheless, the viral titer in saliva is sufficient for SARS-CoV-2 detection^34^. To adapt eRPA to saliva samples, we tested protocols that used TCEP, EDTA, and heat steps^23,24^. The addition of the reducing agent TCEP was critical to decreasing the viscosity of saliva at all temperatures, but the inhibition of RNase activity by TCEP was not complete until the sample was heated above 85 °C (Fig. 3e). Because SARS-CoV-2 viral particles lyse at around 75 °C^25^, the period when the sample is being heated from 75 to 85 °C offers a window in which released viral RNA might be degraded during sample preparation. Indeed, RNaseAlert is completely degraded even in the presence of 100 mM TCEP if it is added before the heat inactivation step, but protected if it is added after heat inactivation (Supplementary Fig. 3d, e). This distinction is critical as a common method of validating extraction-free saliva sample preparation protocols is to first heat-inactivate the sample and then add viral RNA to determine assay sensitivity^15^. This method will overestimate assay sensitivity for saliva samples due to the inactivation of salivary RNases. Either murine RNase inhibitor or RNasin Plus helped protect RNA from degradation at low temperatures, with RNasin Plus being more effective at high temperatures (Supplementary Fig. 3f). The combination of RNasin Plus and TCEP protects RNaseAlert from degradation during a heat lysis protocol (Fig. 3f and Supplementary Fig. 3g). Using this protocol (Fig. 3g) we detected SARS-CoV-2 signal in ~70% of samples with 25–100 AccuPlex viral particles in saliva (Fig. 3h), a reduction of 2 to 4-fold compared to the sensitivity of detection in VTM (Fig. 3d). We saw similar results with IVT SARS-CoV-2 RNA which represents the worst-case scenario for RNA degradation (Fig. 3h). Given that titers of SARS-CoV-2 in saliva are in the range of 10^4^ to 10^10^ copies per mL^34^, this extraction protocol combined with eRPA should be able to identify COVID-19 in a high proportion of infected patients, offering the potential for a high throughput, first pass screening approach that could be important in large-scale testing. We note that we have not yet tested eRPA on actual saliva samples from infected individuals, as these are not readily available.

### Comparison of eRPA with RT-qPCR tests on unextracted clinical samples

**Fig. 4:**
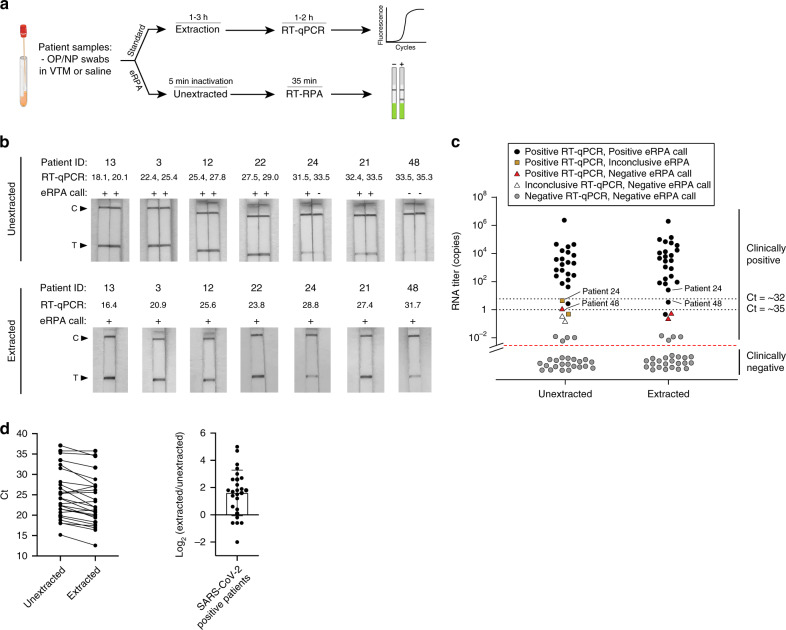
Detection of SARS-CoV-2 in clinical samples using eRPA. **a** Schematic of the workflow for benchmarking eRPA against RT-qPCR using patient samples. **b** Sampling of lateral flow strip readouts of SARS-CoV-2 N gene eRPA tests of unextracted (top) or extracted (bottom) patient samples (*n* = 7 biologically independent samples) of known infection status. Unextracted patient samples were run in duplicates both by eRPA (calls of positive (+) or negative (−) were made within 20 min of detection) and by one-step RT-qPCR (Ct values shown). See additional data in Supplementary Fig. 4a. RNA was extracted from clinical samples according to standard procedure (see “Methods” section) and was subsequently used as input to eRPA and RT-qPCR. See additional data in Supplementary Fig. 4b. **c** Summary of eRPA test results of patient samples (*n* = 51 biologically independent samples) and comparison to RT-qPCR. The *y* axis represents patient viral titer determined using a commercial one-step RT-qPCR assay from unextracted samples or extracted RNA samples with a standard curve. **d** (Left) Matched RT-qPCR Ct values of unextracted and extracted patient samples (*n* = 26 biologically independent samples) (Right) Difference between extracted and unextracted Ct values for all patients (*n* = 26 biologically independent samples) with mean value of 1.6 fold +/− 1.7 SD. Source data are available in the Source Data file.

To demonstrate that eRPA can detect SARS-CoV-2 in unextracted patient samples, we obtained 30 positive and 21 negative NP swabs from BocaBiolistics (Supplementary Data 3). We processed the samples using our VTM heat lysis method (Fig. 3c) and used this unextracted input, in parallel, in eRPA and in a one-step RT-qPCR assay (Fig. 4a). We validated our one-step RT-qPCR assay by benchmarking it against the standard CDC N1 RT-qPCR assay (Supplementary Fig. 4, see Methods). All 21 negative samples were negative by eRPA, in duplicate, confirming that the false positive rate for eRPA is very low (Supplementary Fig. 4b, c and Supplementary Data 3). Of the 30 positive samples, 26 had signal by RT-qPCR; 4 may have suffered degradation during transit, see below. For each of the 26 samples that were positive by RT-qPCR, we estimated the number of copies of input RNA into eRPA based on standard curves (Supplementary Data 3). Twenty samples had an input of at least five molecules of RNA; all 20 of these were positive by eRPA in two repeats (Supplementary Fig. 4a). In three samples the input was between one and four copies; eRPA was positive once, inconclusive once (one positive and one negative of two replicates), and negative once. In three samples the input was less than one copy, and two of these three samples were inconclusive by RT-qPCR; eRPA was negative twice and inconclusive once (Fig. 4c).

We repeated the eRPA workflow on the S gene and obtained similar results to the N gene (Supplementary Fig. 5). In all the patient samples, the RNA copy number of the S gene was on average 4-fold lower than that for the N gene (Supplementary Fig. 5b). This is puzzling, as the detected copy number for both genes are nearly identical from synthetic full genome SARS-CoV-2 RNA, AccuPlex viral particles, and IVT RNA controls (Supplementary Fig. 5b–d). One possible explanation for this result is that viral transcripts from patient cells present in the NP swab contribute significantly to the total RNA detected. Because the N gene is expressed at up to 10-fold higher levels than the S gene in infected human cells^35^, if NP swabs collect cells or cellular debris this would bias the observed N gene to S gene copy number ratio. This may be important for other assays as many COVID tests target ORF1ab, which is one of the lower expressed transcripts in human cells.

To validate the results of eRPA and the RT-qPCR results from unextracted samples, we performed RNA extraction on all samples and then repeated RT-qPCR and eRPA (Fig. 4a). Overall, RNA extraction increased RNA titer by ~5-fold, matching expectations given that 240 µL of initial sample was concentrated into 50 µL of final volume (Supplementary Fig. 4b–d). eRPA gives concordant results with RT-qPCR in all extracted samples except those with extremely low titer. Of the 26 extracted samples that were detected as positive by RT-qPCR without extraction, 23 had at least 3 copies of input RNA, and all of these were positively identified by eRPA (Supplementary Fig. 4b). Three samples had <1 copy of input RNA, of which one was identified by eRPA. The four samples with undetectable signal by RT-qPCR before extraction were still negative by both RT-qPCR and eRPA even with extraction. We note that modest changes in sample collection methods would make clinical diagnostics even more sensitive. Currently, NP swabs are typically collected into 3 mL of VTM. Only a small fraction of this volume is used for detection assays. If instead swabs were resuspended in 150–200 µL of liquid, the volume required to cover the head of a swab, the input would become ~15–20-fold more concentrated without requiring an extraction protocol. This could make the sensitivity of eRPA superior to the current clinical standard of sample extraction followed by RT-qPCR.

## Discussion

The eRPA protocol reported here was developed and optimized in just under 3 weeks, with an additional 4 weeks for sample preparation optimization, and patient sample acquisition. In future epidemics and pandemics, this process could be shortened to several days after standardizing sample preparation methods and primer design, and streamlining IRB and COMS approvals. eRPA addresses many of the problems of current SARS-CoV-2 testing methods: it is scalable, compatible with both swabs and saliva samples, can be performed in parallel by minimally trained personnel in low-resource settings (Supplementary Fig. 6). A companion manuscript shows that the improvements in RT-RPA we implemented in eRPA also improve other detection approaches such as SHINE^36^, allowing these assays to become 1-pot, closed-tube, fluorescent readout reactions. eRPA brings us closer to an at-home SARS-CoV-2 test and continued efforts to improve eRPA include: (i) making reactions self-contained and not requiring the addition of reagents before readout, and (ii) making the assay readout quantitative by integration with a lateral flow reader or smartphone app. eRPA is capable of reliably detecting SARS-CoV-2 virus in patient samples that contain as low as five viral particles, and is therefore fully adequate to detect infection during the period of peak transmission^27–30,37,38^.

## Methods

### RNA template preparation

SARS-CoV-2, SARS-CoV, and MERS N gene containing plasmids were obtained from IDT (2019-nCoV Plasmid Controls). HCoV-229E and HCoV-HKU1 N gene, SARS-CoV-2, SARS-CoV, MERS, HCoV-229E, and HCoV-HKU1 S gene were synthesized by Twist Bioscience. All genes were cloned into a T7 promoter expression plasmid. To produce RNA template, in vitro transcription was performed with NxGen® T7 RNA Polymerase (Lucigen #F88904-1) according to the manufacturer’s suggested protocol with minor modifications. Final concentrations of the reaction mixture components were 50 units T7 RNA polymerase, 1× reaction buffer, 625 µM NTPs, 10 mM DTT, 500 ng of linearized plasmid template, and RNase-free water to a final volume of 20 µL per reaction. After 10 h at 37 °C, four units of DNase I (NEB #M0303S) was added and reactions were further incubated for 10 min at 37 °C. DNase I was heat inactivated by adding EDTA (5 mM final) and heating at 75 °C for 10 min. RNA was purified by RNAClean XP (Beckman Coulter) at 0.6× the volume of the reaction, washed twice with 80% EtOH, then eluted into 20 µL RNase-free water. The size and quality of the RNA product was checked by Bioanalyzer (Agilent) after denaturation at 70 °C for 2 min to unfold any RNA structure; all samples were determined to contain the correct RNA product.

To quantify the concentration, each RNA stock was serially diluted in nuclease-free water down to 0.005 molecules/µL and 2 µL was used as input to the RT-qPCR. Poisson distribution based on Ct values of serially diluted samples was used to calculate the stock concentration.

### Reverse transcription and RNase H screen

eRPA assay master mixes targeting SARS-CoV-2 N gene were prepared as described below with or without addition of RNase H (NEB) and without reverse transcriptase. The following RT enzymes were added to aliquots of the master mixes: SuperScript III (ThermoFisher), SuperScript IV (ThermoFisher), MMLV (Moloney Murine Leukemia Virus RT, NEB), ProtoScript II (NEB), Maxima H Minus RT (ThermoFisher). All enzymes were added at 20 U per reaction. N gene IVT RNA diluted with H_2_O was used as input to the reactions. Post isothermal amplification, samples were diluted 1:400 in water and products were detected by qPCR using primers JQ289 and JQ223. Specific products were distinguished from primer dimers by analyzing the melting temperature of the qPCR products. The average Ct value of all water control reactions representing primer dimer was used as a baseline to determine the reaction yield (yield = Ct (average water controls) – Ct (specific reaction)).

### Primer oligomerization products

Four N gene forward primers (JQ217, CCMS041, CCMS047, and CCMS051) were paired with the reverse primer JQ224. These four primer pairs as well as JQ217 + JQ223 were used in eRPA assays with a water-only sample input. eRPA assays were incubated at 42 °C for 10 min. Amplification products were cleaned up using RNA Clean XP (Beckman Coulter) at 2.5× concentration and eluted in 20 µL of nuclease-free water. Purified products were cloned using the Zero Blunt TOPO PCR Cloning Kit (Thermo Fisher Scientific) according to the manufacturer’s instructions and Sanger sequenced. The identity of cloned products was determined by first aligning the sequences to the vector sequence using Samtools (v1.9) with an allowed multimapping of *k* = 10,000. The sam files were then visualized in IGV (v2.6.2) where the direction, sequence, and copy number of primer oligomers were manually annotated. In Table S2, the direction of the primer indicates a forward or reverse direction with respect to the vector, while the space sequence is the string of nucleotides between two primers. Overlap indicates the primers were overlapping with respect to the vector, “*” indicates there was no space between the primers, and listed nucleotides indicate the sequence between two primers.

### SARS-CoV-2 eRPA assay primer sequence alignment

To calculate the percent identity between the SARS-CoV-2 N and S gene primers and the analogous sites in other betacoronaviruses, the RefSeq entries for SARS-CoV-2, SARS-CoV, MERS, HCoV-229E, HCoV-NL63, HCoV-OC43, and HCoV-HKU1 were obtained from NCBI. The sequences were then compared using the EMBL-EBI web tool Clustal Omega to identify indels and mismatches. The subsequences for the forward and reverse primers for both the N gene and the S gene were then located within the SARS-CoV-2 sequence, and the number of mismatches with the antagonist betacoronavirus sequence was tallied. The percent identity was then calculated by dividing the number of matching bases by the length of the primer sequence.

To calculate the number of mismatches between eRPA assay primer and probe sequences and known SARS-CoV-2 variants, the full set of all available SARS-CoV-2 genomes were downloaded from NCBI and were arranged into a single fasta file. This dataset was then converted into a BLAST database using the BLAST + (v2.6.0) tool and then queried by each of the sequences for the N and S gene eRPA assay. The output from BLAST was then coalesced and filtered to remove any incomplete or partial genomes using R (v.4.0).

### Primer screening

Regions of low homology between SARS-CoV-2 and both SARS-CoV and MERS were identified by sequence alignment and were used as target sequences for the biotinylated probe. Unlabeled forward and reverse primers (Supplementary Table 1) were designed to amplify a region of 100–200 nt encompassing the target sequence. Combinations of forward and reverse primers were screened by testing RPA amplification at low RNA input. Reactions were prepared as described below and S gene IVT SARS-CoV-2 RNA was used as input. Post amplification, samples were diluted 1:625 in water and products were detected by qPCR using the same primer pair as for RPA amplification. Specific products were distinguished from primer dimers by analyzing the melting temperature of the qPCR products. Reactions using water only as template were used to identify primer dimer melting temperatures. All reactions with 10 or 100 copies input but leading only to the formation of primer dimers were labeled as having a reaction yield of zero. The Ct of all reactions leading to specific product formation were converted into an estimated reaction yield by subtracting the raw Ct from the Ct of the lowest specific reaction (for the S gene screen the lowest specific Ct was at 25). Primer pairs with high reaction yields at both 100 and 10 copies input were tested in a secondary screen and the top two primer pairs were subsequently tested by eRPA.

### eRPA assay

Isothermal amplification reactions were based on the TwistAmp Basic RPA Kit (TwistDx) with added modifications described below. Each lyophilized pellet was resuspended in a solution of 38 µL rehydration buffer (TwistDx), 1 µL RNase H (5U/µL) (NEB), 0.5 µL SuperScript IV RT (200 U/µL) (ThermoFisher Scientific), and 0.5 µL of forward and reverse primer mix each at 50 µM (N gene, JQ217 + JQ235; S gene, CCMS055 + CCMS073). This mix was then activated by addition of 1 µL 700 mM magnesium acetate followed by thorough mixing with a pipette. Reactions were prepared by dispensing 8 µL of master mix and 2 µL of input template (RNA, Accuplex virus, or patient samples) per reaction well, mixing the reaction by pipetting, and incubating at 42 °C for 25 min. A hybridization mix was prepared by combining 1 µL biotinylated probe at 5 µM (N gene, JQ241 or JQ312; S gene, CCMS069) with 19 µL 10 mM Tris pH 8. 20 µL of hybridization mix was added to each reaction, and samples were heated at 94 °C for 3 min followed by a cooling step at room temperature for 3 min. 50 µL of Milenia GenLine Buffer (Milenia Biotec) was added to each reaction, mixed by pipetting, and a lateral flow strip (Milenia HybriDetect) was added. Lateral flow strip signals can be detected and imaged starting 3 min after addition of the strip to the hybridized reaction. Test results were called or imaged within 30 min of strip addition since background bands at the test line can appear over time and low signal test bands can lose intensity as the strip dries.

### qPCR and RT-qPCR

SYBR green qPCR reactions were prepared in 10 µL reaction volume using PowerUp SYBR Green PCR Master mix (Thermo Fisher Scientific), 2 μL sample, and 0.4 μM of primers (JQ217 + JQ223 for N gene or CCMS055 + CCMS067 for S gene unless otherwise mentioned). RT-qPCR reactions were prepared in 10 µL reaction volume using the Luna Universal One-Step RT-qPCR kit (NEB), 2 μL sample, and 0.4 μM of primers following the manufacturer’s instructions. The CDC one-step RT-qPCR assay used to benchmark our in-house RT-qPCR was performed using the Luna Universal Probe One-step RT-qPCR kit (NEB) and N1 probe/primer mix against SARS-CoV-2 from IDT (2019-nCoV CDC EUA Kit) (Supplementary Fig. 4d). Reactions were prepared according to the manufacturer’s instructions following the CDC protocol. qPCR and RT-qPCR reactions were monitored on either a Bio-Rad C1000 Touch Thermo Cycler (Bio-Rad) or QuantStudio 6 Real Time PCR system (Thermo Fisher Scientific).

### Sensitivity and specificity of eRPA with RNA input

Data presented in Fig. 2 was generated as a blinded and randomized experiment. Synthetic full genome SARS-CoV-2 RNA (Twist Bioscience) was used as RNA template for eRPA assay on SARS-CoV-2. For the cross-reactivity samples, a single dilution series of RNA input was prepared by mixing at equimolar ratio N and S gene IVT RNA products for each of: SARS-CoV, MERS, HCoV-HKU1, and HCoV-229E. Genomic 2009 H1N1 Influenza (ATCC) was also serially diluted for input to the assay. All dilutions series were made in water and were adjusted for a 2 µL input into the eRPA assay. Two independent groups prepared fully randomized 96-well PCR plates in a checkerboard pattern using those dilutions (Supplementary Fig. 2a). Each group then used the other group’s randomized plate as input to eRPA tests targeting either the N gene or the S gene of SARS-CoV-2 performed as described above. All RNA stocks used in these tests were validated by testing dilution series in a one-step RT-qPCR as described above (Fig. 2b and Supplementary Fig. 2d–f).

### RNaseAlert tests with viral transport media (VTM) and saliva

The RNaseAlert substrate (IDT) was used at 2 µM to assess the RNase activity of saliva and VTM (BD, universal viral transport medium #220220). Fluorescence intensity was determined using an excitation of 485 nm and emission of 528 nm over the course of 10–60 min in a 96-well plate reader (Synergy H1 Plate Reader, BioTek). In general, the degradation of the RNaseAlert substrate was assessed after 10 min and fluorescence intensities were averaged over three time points and reported normalized to a fully degraded control.

RNasin Plus (Promega) was added to VTM to a final concentration of 1 U/µL and was incubated for 5 min at 25 °C before addition of RNaseAlert. When needed, viral lysis buffer (FastAmp Viral and Cell solution, Intact Genomics) was added 1:1 (v/v) to VTM. TCEP buffer (20 mM Tris pH 8, 10 mM EDTA pH 8, TCEP 1–100 mM) was prepared as a 2× solution and was mixed 1:1 with saliva. RNase inhibitor (RI) was added to 1 U/µL final concentration as shown. For spike-in controls, RNase A (Lucigen) was added to 0.25 µg/µL final concentration. Saliva obtained from two healthy donors was pooled and adjusted to 1 mM TCEP to reduce viscosity. Aliquots of a single pooled sample stored at −20 °C were used for all assays. Saliva samples were obtained from volunteers as approved by the Harvard Medical School Institutional Review Board (IRB 20-0581). Informed written consents were obtained by volunteers.

### Virus extraction

The AccuPlex SARS-CoV-2 verification panel (Seracare) containing the N gene, E gene, ORF1a, and RdRp was used as a surrogate to SARS-CoV-2 to optimize the full processing of clinical samples. To determine the temperature lysis of AccuPlex SARS-CoV-2, virus at 1e5 copies/mL was diluted 1:1 in 2× lysis buffer (final: 10 mM Tris HCl pH 8, 5 mM EDTA pH 8, 100 mM TCEP, 1 U/µL RNasin Plus), then incubated for 5 min at a temperature between 55 and 95 °C in 5 °C increments. 2 µL of each condition was used as input into eRPA reactions targeting SARS-CoV-2 N gene as described above. Postamplification, samples were diluted 1:200 in water and products were detected by qPCR using primers JQ289 and JQ223. Specific product formation was distinguished from primer dimer formation by analyzing the melting temperature of the qPCR products and comparison to a water control. The average Ct value of all water control reactions representing primer dimer was used as a baseline to determine the reaction yield (yield = Ct (average water controls) – Ct (specific reaction)).

### Detection of AccuPlex SARS-CoV-2 in contrived samples

AccuPlex SARS-CoV-2 was extracted using conditions mimicking patient sample processing. eRPA assays targeting SARS-CoV-2 N gene were performed as above. For extraction in VTM and PBS, AccuPlex SARS-CoV-2 at 100 copies/µL was serially diluted 1:1 (v/v) in either VTM or PBS containing a final concentration of 1 U/µL RNasin Plus. After heating at 94 °C for 5 min, samples were kept on ice before being used as input into eRPA. For extraction in viral lysis buffer at 25 °C, AccuPlex SARS-CoV-2 at 100 copies/µL was serially diluted 1:1 (v/v) in viral lysis buffer (FastAmp Viral and Cell solution (Intact Genomics)) adjusted with RNasin Plus to 1 U/µL. After 10 min at 25 °C, samples were kept on ice before being used as input into eRPA. For extraction of virus in samples containing saliva, 2 vol of AccuPlex SARS-CoV-2 virus at 100 copies/µL was mixed with 1 vol of pooled saliva and 1 vol of 4× TCEP buffer + RI. TCEP buffer + RI was prepared such that final buffer concentrations in the sample were 10 mM Tris HCl pH 8, 5 mM EDTA pH 8, 100 mM TCEP and 1 U/µL RNasin Plus. Lower input samples were prepared by serial dilution with 1:1 (v/v) saliva in 2× TCEP buffer. After heating at 94 °C for 5 min, 1/10 vol of 1 M H_2_O_2_ was added and samples were incubated at 25 °C for 10 min. Saliva samples were diluted 1:1 with water and kept on ice before being used as input into eRPA. For extraction of virus from saliva with viral lysis buffer, 1 vol of AccuPlex SARS-CoV-2 virus at 100 copies/µL was mixed with 1 vol of pooled saliva and 2 vol of viral lysis buffer adjusted to 2 U/µL RNasin Plus. Lower input samples were prepared by serial dilution with 1:3 (v/v) viral lysis buffer + RI mixed with saliva. For samples with SARS-CoV-2 RNA, saliva was mixed 1:1 with 2× TCEP buffer + RI. After 5 min at 25 °C, N gene IVT SARS-CoV-2 RNA was spiked into saliva in TCEP buffer and lower input samples were prepared by serial dilution on ice. After heating at 94 °C for 5 min, 1/10 vol of 1 M H_2_O_2_ was added and samples were incubated at 25 °C for 10 min. Samples were diluted 1:1 with water and kept on ice before being used as input into eRPA. For RNA added post heat inactivation, a similar protocol was followed using saliva mixed 1:1 with 2× TCEP buffer + RI that was preincubated for 5 min at 94 °C.

### Clinical samples

A cohort of nasal swab patient samples was purchased from BocaBiolistics, FL containing 30 SARS-CoV-2 positive samples and 21 SARS-CoV-2 negative samples. Samples were thawed on ice and 40 µL aliquots were made and subsequently stored at −80 °C. At the time of testing, sample aliquots were thawed and RNasin Plus was added to a final concentration of 1 U/µL. The samples were placed on a heat block set to 99 °C for 5 min for virus inactivation and lysis. After cooling, samples were spun down and transferred to a 96-well DNA LoBind plate (Eppendorf). Two microliter of the inactivated sample was used as input into eRPA or into RT-qPCR reactions targeting both the N and S gene of SARS-CoV-2 (Supplementary Table 4). GAPDH was used as a control in RT-qPCR reactions. All patient sample tests included a positive control consisting in 100 copies of synthetic full genome SARS-CoV-2 RNA (Twist Bioscience) and a water only negative control.

### Standard RNA extraction from clinical samples

Virions were pelleted by centrifugation at approximately 21,000×*g* for 2 h at 4 °C. The supernatant was removed and 750 µL of TRIzol-LS™ Reagent (ThermoFisher) was added to the pellets and then incubated on ice for 10 min. Following incubation, 200 µL of chloroform (MilliporeSigma) was added, vortexed, and incubated on ice for 2 min. Phases were separated by centrifugation at 21,000×*g* for 15 min at 4 °C, and subsequently the aqueous layer was removed and treated with 1 vol isopropanol (Sigma). GlycoBlue™ Coprecipitant (15 mg/mL) (ThermoFisher) and 100 µL 3 M Sodium Acetate (Life Technology) were added to each sample and incubated on dry ice until frozen. RNA was pelleted by centrifugation at 21,000×*g* for 45 min at 4 °C. The supernatant was discarded and the RNA pellet was washed with cold 70% ethanol. RNA was eluted in 50 µL of DEPC-treated water (ThermoFisher).

### Quantitative SARS-CoV-2 RT-qPCR assay

Levels of SARS-CoV-2 RNA in extracted samples were detected using the US CDC 2019-nCoV_N1 primers and probe set. Each reaction contained extracted RNA, 1× TaqPath^TM^ 1-Step RT-qPCR Master Mix, CG (ThermoFisher), 500 nM of each the forward and reverse primers, and 125 nM of probe. Viral copy numbers were quantified using N1 qPCR standards to generate a standard curve. The assay was run in triplicate for each sample and two no template control (NTC) wells were included to confirm there was no contamination. Quantification of the Importin-8 (IPO8) housekeeping gene RNA level was performed to determine the quality of sample collection. An internal virion control (RCAS) was spiked into each sample and quantified to determine the efficiency of RNA extraction and qPCR amplification.

In-house RT-qPCR data was converted from Ct values to copies/mL by direct comparison to the CDC RT-qPCR quantitation. In short, the Ct values from the in-house RT-qPCR were plotted against the CDC RT-qPCR Ct values which yielded a linear relationship, *R*^2^ > 0.99, with a slope within error of 1, confirming that the amplification dynamics of both primer sets were similar. The relationship was then refit with the slope set to 1 which yielded a line, *R*^2^ > 0.99, with an intercept of between 33 and 34 (95% confidence interval). This fit was then used to directly convert Ct from the in-house qPCR to viral copies/µL.

### Reporting summary

Further information on research design is available in the Nature Research Reporting Summary linked to this article.

## Supplementary information

Description of Additional Supplementary Files

Supplementary Information

Reporting Summary

Supplementary Data 1

Supplementary Data 2

Supplementary Data 3

## Data Availability

The authors declare that all data generated or analyzed during this study are included in this published article and its Supplementary Information files. A Source Data file is available. Any other relevant data is available upon reasonable request.

## Source data

Source Data
