# Supplementary material for: An enhanced isothermal amplification assay for viral detection
Source: Nat Commun. 2020 Nov 20;11:5920. doi: 10.1038/s41467-020-19258-y (PMC7679446; doi:10.1038/s41467-020-19258-y)
Supplement: Supplementary file 1 — Description of Additional Supplementary Files [file 41467_2020_19258_MOESM1_ESM.pdf]

Title: Supplementary Data 1.

Description: Analysis of primer dimers in RT-RPA reactions.

Title: Supplementary Data 2

Description: Bioinformatic analysis of the number of mismatches between eRPA assay primers and known variants of SARS-CoV-2. Bioinformatic analysis of the number of mismatches between eRPA assay primers and other coronaviruses.

Title: Supplementary Data 3.

Description: Data for all patient sample RT-qPCR and eRPA assays performed in this study
