# Supplementary material for: An enhanced isothermal amplification assay for viral detection
Source: Nat Commun. 2020 Nov 20;11:5920. doi: 10.1038/s41467-020-19258-y (PMC7679446; doi:10.1038/s41467-020-19258-y)
Supplement: Supplementary file 2 — Supplementary Information [file 41467_2020_19258_MOESM2_ESM.docx]

Supplementary Information for

An enhanced isothermal amplification assay for viral detection

Jason Qian^*^, Sarah A. Boswell^*^, Christopher Chidley^*^, Zhi-xiang Lu^*^, Mary E. Pettit, Benjamin L. Gaudio, Jesse M. Fajnzylber, Ryan T. Ingram, Rebecca H. Ward, Jonathan Z. Li, Michael Springer^†^

* These authors contributed equally to this work.

† Corresponding author. Email: michael_springer@hms.harvard.edu

**This PDF file includes:**

Supplementary Figures 1-6

Supplementary Table 1

**Other Supplementary Materials for this manuscript include the following:**

Supplementary Data 1-3

**
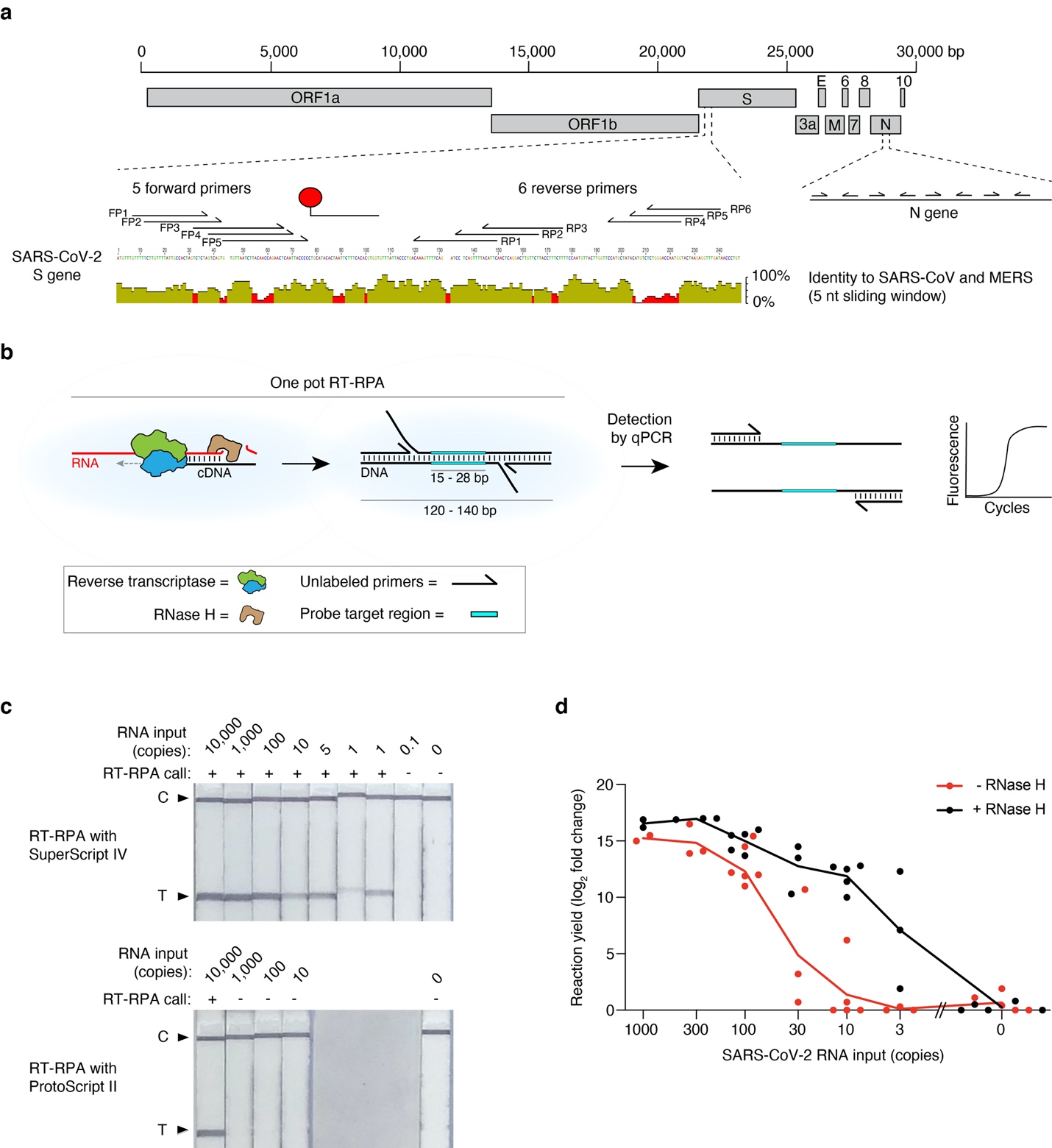
Supplementary Figure 1. Development of eRPA. a** Organization of the SARS-CoV-2 genome and location of regions in the S and N genes targeted by eRPA. Detailed mapping of the binding site of all forward and reverse primers tested in the primer optimization screen and of the biotin hybridization probe was shown for S gene only for display purposes. SARS-CoV-2 was aligned to the closely related SARS-CoV and MERS to identify regions of low homology which were targeted by primers and hybridization probes used in the assay. **b** Schematic of the workflow used for optimization of eRPA. The cDNA product amplified by recombinase polymerase amplification (RPA) using forward and reverse unlabeled primers was quantified in a subsequent qPCR assay. **c** Comparison of the performance of SuperScript IV and ProtoScript II. In vitro transcribed (IVT) N gene SARS-CoV-2 RNA was amplified by RT-RPA and reactions were read out on a lateral flow strip. **d** IVT N gene SARS-CoV-2 RNA was amplified by RT-RPA with or without RNase H addition and the yield of each reaction was determined by quantitative PCR. Data represent the average yield of two technical replicates and is staggered on the x axis for visualization purposes.


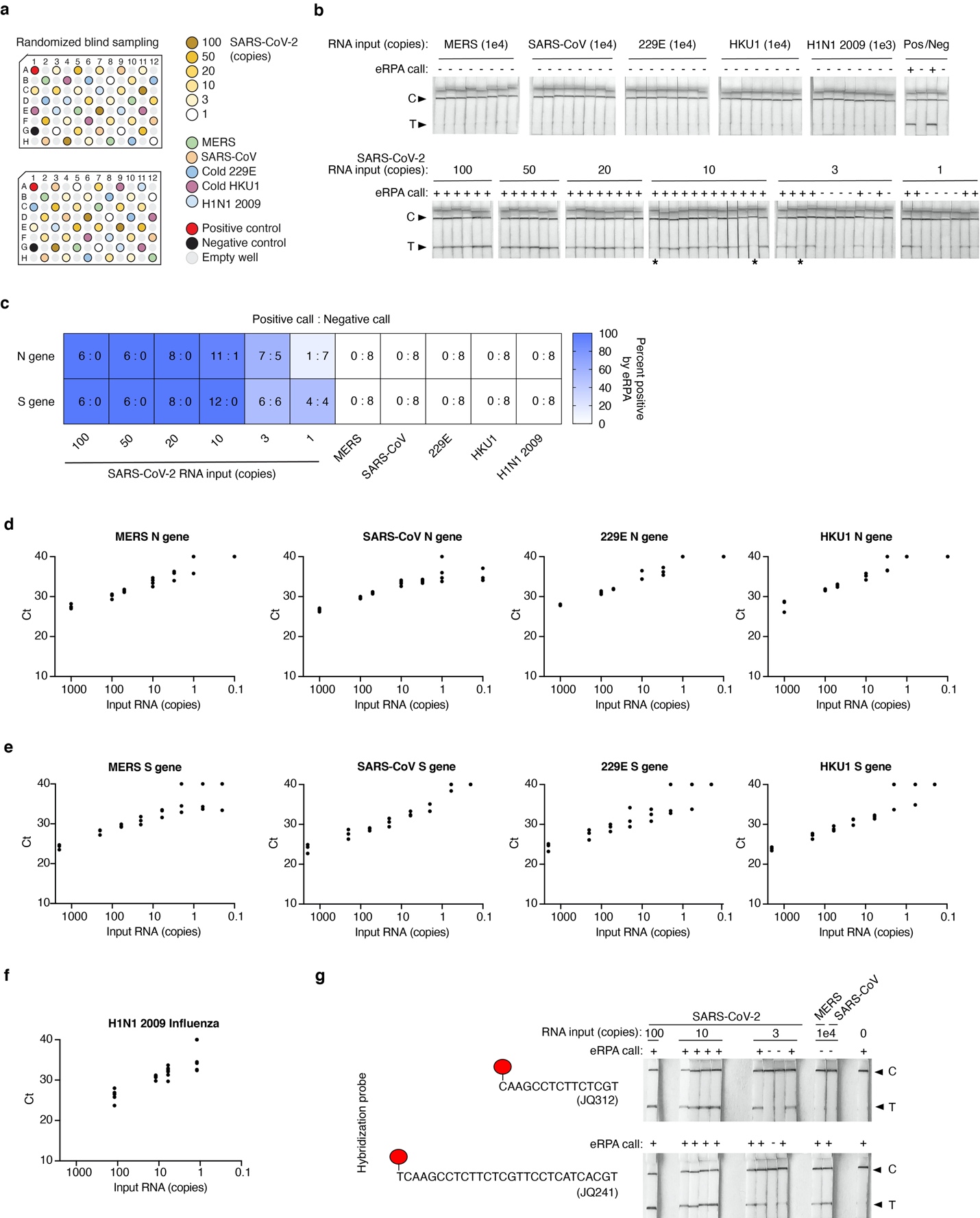


**Supplementary Figure 2. Sensitivity and specificity of RNA detection. a** Blinded and randomized plate layout used in eRPA assays used for generation of the data displayed in **Fig. 2a**. **b** Lateral flow strip readouts for the S gene dataset displayed in **Fig. 2a**. Individual strips are labeled with the test call made within 20 mins of detection (positive (+) or negative (-)). The positive (Pos.) eRPA control is 1,000 copies of synthetic full genome SARS-CoV-2 RNA and the negative (Neg.) eRPA control is a water-only input. Images taken for the purpose of display were allowed to dry which reduced the intensity of some weak bands (labeled with asterisks). **c** Heatmap displaying the rate of eRPA test calls for detection of RNA from SARS-CoV-2 or from other viruses as shown in **Fig. 2a**. Values represent the number of positive test calls : number of negative test calls for each condition. **(d-e)** RT-qPCR quantification of in vitro transcribed (IVT) RNA from MERS, SARS-CoV, HCoV-229E, and HCoV-HKU1 used as specificity control tests in eRPA; N gene shown in **d** and S gene in **e**. **f** RT-qPCR quantification of RNA extracted from 2009 H1N1 Influenza. **g** Comparison of the specificity and sensitivity of two hybridization probes targeting SARS-CoV-2 N gene. IVT RNA from SARS-CoV-2, MERS, or SARS-CoV was amplified by eRPA. After splitting the reactions in half and hybridizing with a biotinylated probe as shown, each reaction was read out on a lateral flow strip. Individual strips are labeled with the test call made within 20 mins of detection (positive (+) or negative (-)).

**
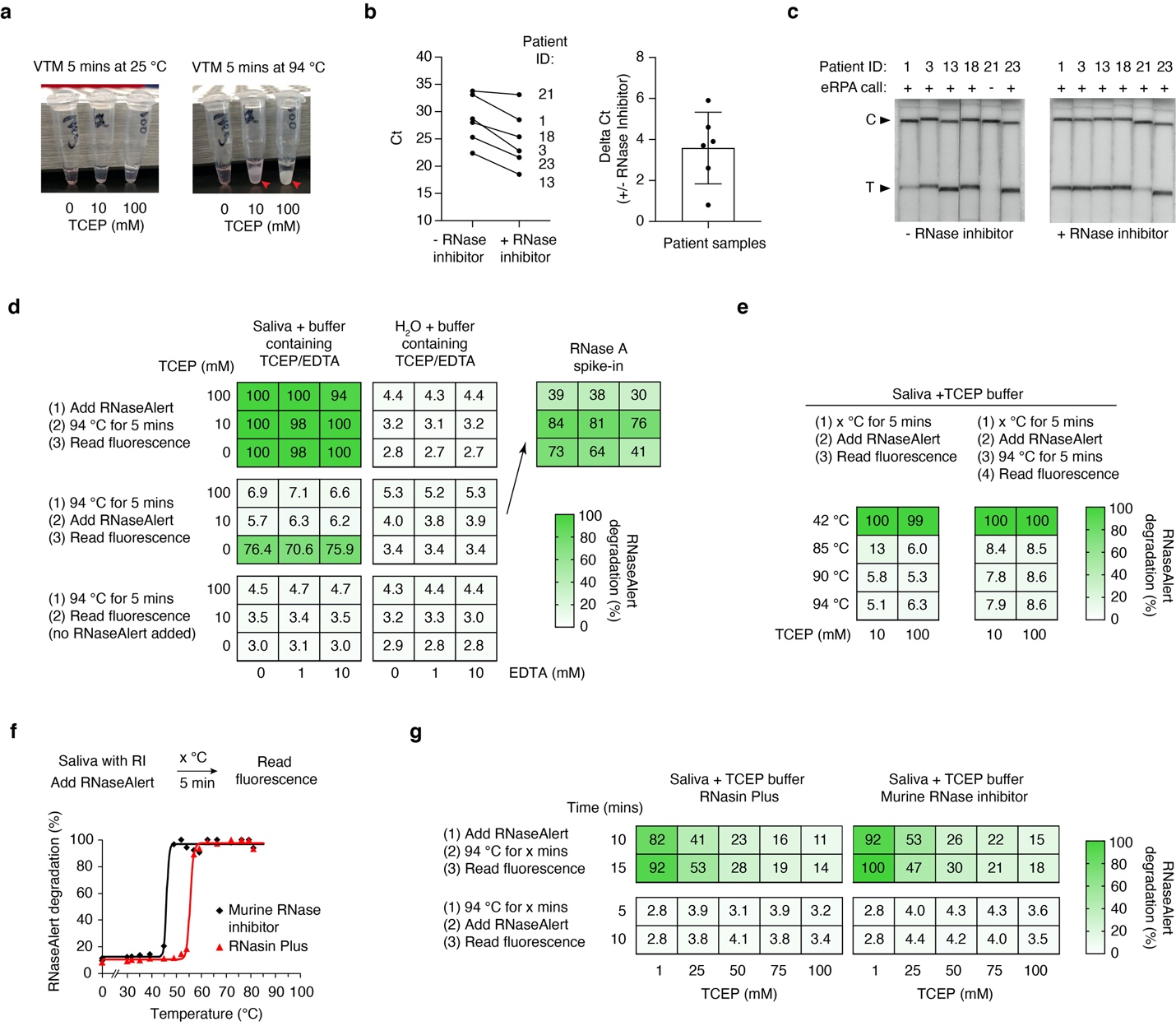
Supplementary Figure 3. Optimization of sample processing conditions for detection of SARS-CoV-2 in clinical samples. a** Heating VTM in presence of TCEP leads to formation of a gelatinous substance (highlighted by red arrowhead). **b** Addition of RNase inhibitor to patient samples prior to heat inactivation increases the RNA titer as quantified by RT-qPCR. Unextracted known positive patient samples were heat inactivated for 5 min at 94°C with or without RNasin Plus. Viral RNA was quantified using a commercial one-step RT-qPCR assay. (Left) Ct values for matched samples (n=6 biologically independent samples) with and without RNase inhibitor. (Right) Difference between Ct values in all matched samples (n=6 biologically independent samples) with mean value of 3.6 fold +/- 1.7 SD. Error bars represent +/- 1 standard deviation. **c** Addition of RNase inhibitor to patient samples prior to heat inactivation increases the signal of the eRPA assay. Heat inactivated samples prepared in **b** were tested using eRPA. **d** TCEP and heat (not EDTA) are required to inactivate the RNase activity in saliva as determined using RNaseAlert assays. Saliva (or water control) was mixed 1:1 with a buffer containing TCEP and EDTA as shown. RNaseAlert was added and the sample was heated as indicated. RNase A was added to a set of water samples post addition of RNaseAlert as control. Data represent the average of 2 technical replicates and was determined from the fluorescence signal 10 mins after the heating step normalized to a fully degraded control. **e** TCEP and heat irreversibly inactivate the RNase activity of saliva. Saliva was mixed 1:1 with a buffer containing TCEP and was processed as indicated. Data represent the average of 3 technical replicates and was determined as in **d**. **f** RNase inhibitors protect RNA against degradation in saliva at low temperature only. Saliva was mixed 1:1 with a buffer containing an RNase inhibitor as shown. RNaseAlert was added and the sample heated as indicated. Data represent the fluorescence intensity 10 mins after the heating step normalized to a fully degraded control. **g** The combined activities of an RNase inhibitor and TCEP protect RNA from degradation in saliva (Additional data for **Fig. 3f**).

**
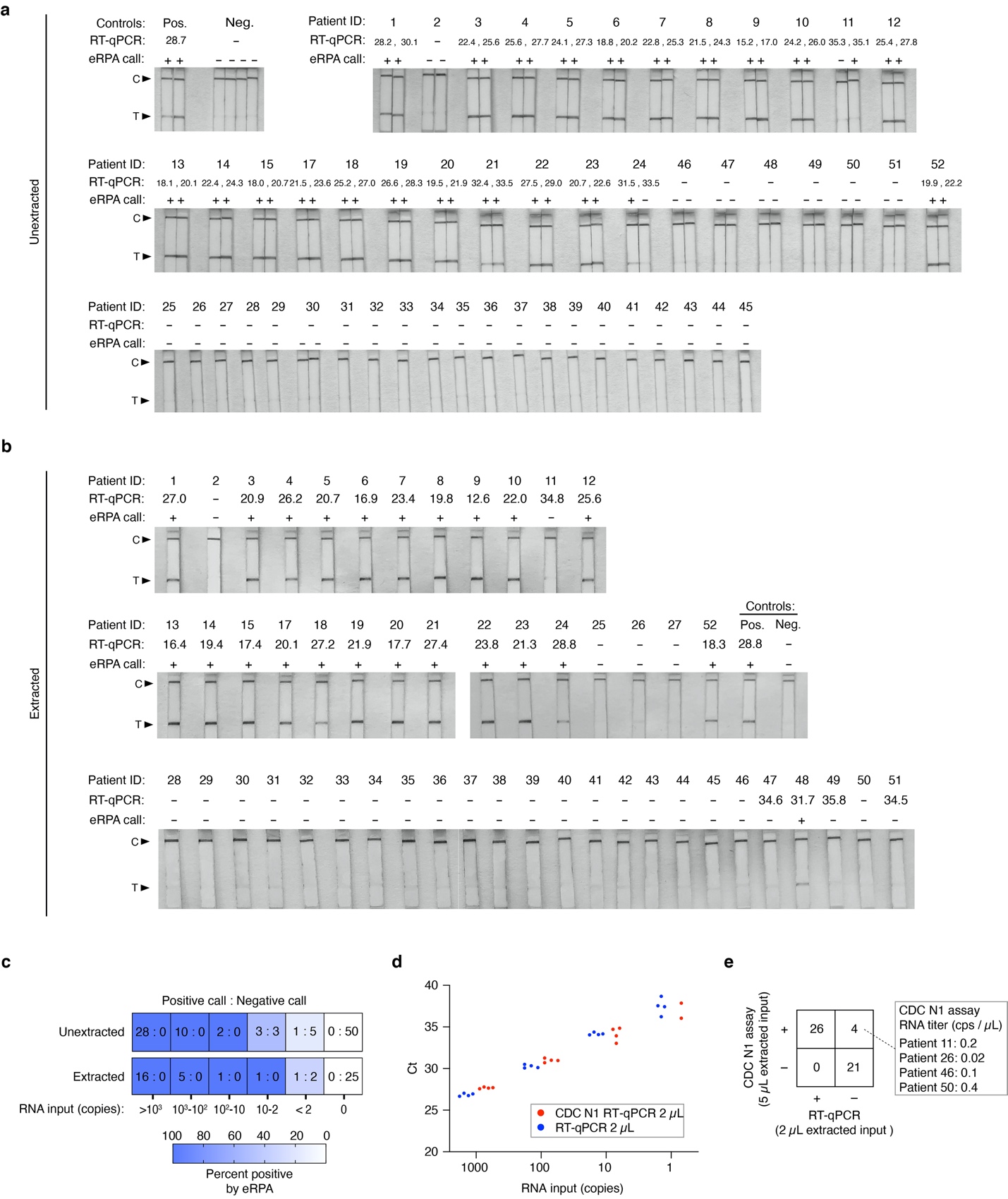
**

**Supplementary Figure 4. Detection of SARS-CoV-2 from unextracted and extracted clinical samples. (a-b)** Lateral flow strip readouts of all eRPA tests from unextracted (**a**) and extracted (**b**) patient samples summarized in **Fig. 4d**. Individual strips are labeled with the eRPA test call made within 20 mins of detection (positive (+) or negative (-)). The positive (Pos.) eRPA control is 100 copies of synthetic full genome SARS-CoV-2 RNA and the negative (Neg.) eRPA control is a water-only input. **c** Heatmap displaying the rate of positive eRPA test calls for detection of SARS-CoV-2 N gene from the 51 patient samples as shown in **Fig. 4d** binned by RNA input determined by one-step RT-qPCR. Values represent the number of positive test calls : number of negative test calls for each condition. **d** Our one-step RT-qPCR assay was validated against the CDC N1 RT-qPCR assay using synthetic SARS-CoV-2 RNA as input. **e** Comparison between the sensitivity of the CDC N1 RT-qPCR assay ran on 5 μL extracted sample and our one-step RT-qPCR assay ran on 2 μL extracted sample.

**
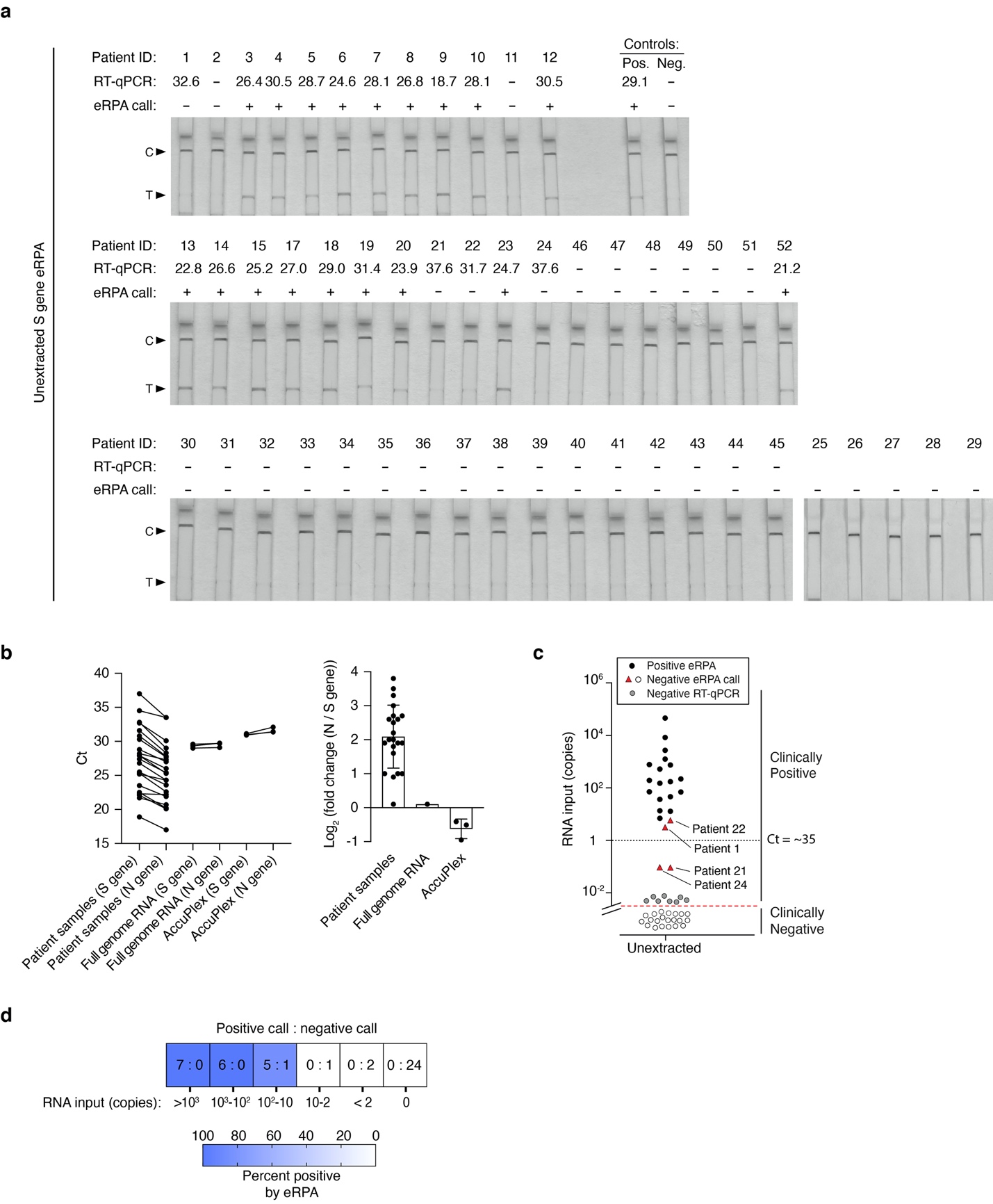
Supplementary Figure 5. Detection of SARS-CoV-2 S gene from clinical samples. a** Lateral flow strip readouts of S gene eRPA performed on patient samples of known infection status. Individual strips are labeled with the test call made within 20 mins of detection (positive (+) or negative (-)). The positive (Pos.) eRPA control is 100 copies of synthetic full genome SARS-CoV-2 RNA and the negative (Neg.) eRPA control is a water-only input. Negative control samples 25-29 were not screened by RT-qPCR. **b** Comparison of Ct values obtained by RT-qPCR targeting SARS-CoV-2 N and S genes on the same input patient samples. (Left) Matched patient samples (n=22 biologically independent samples) are connected by a solid line. Synthetic full genome SARS-CoV-2 RNA (n=3 biologically independent samples) and AccuPlex packaged SARS-CoV-2 (n=3 biologically independent samples) were used as controls as they both contain an equal amount of N and S gene. (Right) Difference between the Ct values for each patient sample (n=22 biologically independent samples) is plotted with mean value 2.1 fold +/- 0.9 SD. For synthetic full genome SARS-CoV-2 RNA the mean value is 0.1 fold; For AccuPlex packaged SARS-CoV-2 the mean value is -0.6 fold +/- 0.3 SD. **(c-d)** Sensitivity and specificity of S gene eRPA on patient samples shown in **a**. **c** Comparison between S gene SARS-CoV-2 eRPA and one-step RT-qPCR performed on the same input samples. The y axis is RNA copies in patient input samples determined by one-step RT-qPCR with standard curve. **d** Heatmap displaying the rate of eRPA positive tests for detection of SARS-CoV-2 S gene in the 51 patient samples. Values represent the number of positive test calls : number of negative test calls for each condition.

**
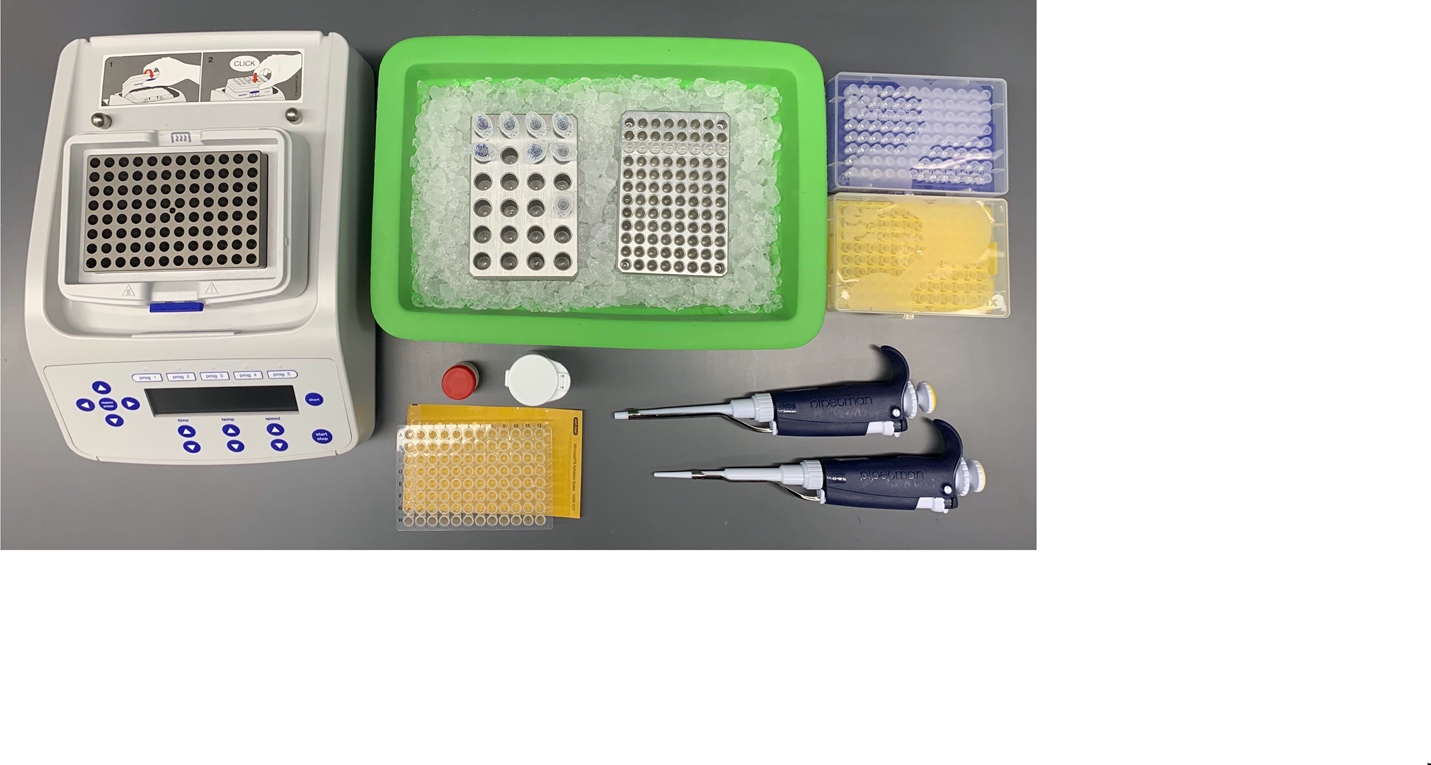
**

**Supplementary Figure 6. Equipment required for eRPA assay.** eRPA only requires a limited set of equipment including micropipettes and disposable plastic tips, a heat block capable of reaching 42°C and 94°C, and plastic microtubes or multi-well plates.

**Supplementary Table 1.** List of all primers used in this study.

**Supplementary** **Data 1. (separate file)**

Analysis of primer dimers in RT-RPA reactions.

**Supplementary** **Data 2. (separate file)**

Bioinformatic analysis of the number of mismatches between eRPA assay primers and known variants of SARS-CoV-2. Bioinformatic analysis of the number of mismatches between eRPA assay primers and other coronaviruses.

**Supplementary** **Data 3. (separate file)**

Data for all patient sample RT-qPCR and eRPA assays performed in this study.
