# Supplementary material for: An enhanced isothermal amplification assay for viral detection
Source: Nat Commun. 2020 Nov 20;11:5920. doi: 10.1038/s41467-020-19258-y (PMC7679446; doi:10.1038/s41467-020-19258-y)
Supplement: Supplementary file 3 — Reporting Summary [file 41467_2020_19258_MOESM3_ESM.pdf]

nature research

Corresponding author(s): Michael Springer

Last updated by author(s): Sep 22, 2020

Reporting Summary

Nature Research wishes to improve the reproducibility of the work that we publish. This form provides structure for consistency and transparency in reporting. For further information on Nature Research policies, see our Editorial Policies and the Editorial Policy Checklist.

Statistics

For all statistical analyses, confirm that the following items are present in the figure legend, table legend, main text, or Methods section.

n/a	Confirmed	
☐	☒	The exact sample size (n) for each experimental group/condition, given as a discrete number and unit of measurement
☐	☒	A statement on whether measurements were taken from distinct samples or whether the same sample was measured repeatedly
☒	☐	The statistical test(s) used AND whether they are one- or two-sided<br>*Only common tests should be described solely by name; describe more complex techniques in the Methods section.*
☒	☐	A description of all covariates tested
☒	☐	A description of any assumptions or corrections, such as tests of normality and adjustment for multiple comparisons
☒	☐	A full description of the statistical parameters including central tendency (e.g. means) or other basic estimates (e.g. regression coefficient) AND variation (e.g. standard deviation) or associated estimates of uncertainty (e.g. confidence intervals)
☒	☐	For null hypothesis testing, the test statistic (e.g. F, t, r) with confidence intervals, effect sizes, degrees of freedom and P value noted<br>*Give P values as exact values whenever suitable.*
☒	☐	For Bayesian analysis, information on the choice of priors and Markov chain Monte Carlo settings
☒	☐	For hierarchical and complex designs, identification of the appropriate level for tests and full reporting of outcomes
☒	☐	Estimates of effect sizes (e.g. Cohen's d, Pearson's r), indicating how they were calculated

Our web collection on statistics for biologists contains articles on many of the points above.

Software and code

Policy information about availability of computer code

Data collection	Coronavirus sequences from NCBI
Data analysis	EMBL-EBI web tool Clustal Omega, BLAST+ (v2.6.0) , Samtools (v1.9), IGV (v2.6.2)

For manuscripts utilizing custom algorithms or software that are central to the research but not yet described in published literature, software must be made available to editors and reviewers. We strongly encourage code deposition in a community repository (e.g. GitHub). See the Nature Research guidelines for submitting code & software for further information.

Data

Policy information about availability of data

All manuscripts must include a data availability statement. This statement should provide the following information, where applicable:

- Accession codes, unique identifiers, or web links for publicly available datasets
- A list of figures that have associated raw data
- A description of any restrictions on data availability

The authors declare that all data generated or analyzed during this study are included in this published article and its Supplementary Information files.

Field-specific reporting

Life sciences study design

All studies must disclose on these points even when the disclosure is negative.

Sample size	Sample size in this study was not calculated. For clinical sample testing 30 positive and 21 negative samples to meet the standard requirements for assay validation for FDA/EUA clinical validation.
Data exclusions	No data was excluded
Replication	A minimum of two replicates were performed for each condition in a dilution or temperature gradient. For limit of detection and specificity 48 negative and 52 positive samples were tested. For clinical sample testing two independent experiments were performed to ensure results were reliable and reproducible.
Randomization	When randomization was used, a single person randomly allocated the RNA samples into a plate layout they chose. A second blinded person performed the detection assay on the samples and scored results before the layout was revealed.
Blinding	When randomization was used, a single person randomly allocated the RNA samples into a plate layout they chose. A second blinded person performed the detection assay on the samples and scored results before the layout was revealed.

Reporting for specific materials, systems and methods

We require information from authors about some types of materials, experimental systems and methods used in many studies. Here, indicate whether each material, system or method listed is relevant to your study. If you are not sure if a list item applies to your research, read the appropriate section before selecting a response.

Materials & experimental systems

n/a	Involved in the study	
☐	☒	Antibodies
☒	☐	Eukaryotic cell lines
☒	☐	Palaeontology and archaeology
☒	☐	Animals and other organisms
☐	☒	Human research participants
☒	☐	Clinical data
☒	☐	Dual use research of concern

Methods

n/a	Involved in the study	
☒	☐	ChIP-seq
☒	☐	Flow cytometry
☒	☐	MRI-based neuroimaging

Antibodies

Antibodies used	Anti-FAM antibody included in Milenia HybriDetect lateral flow kit.
Validation	https://www.milenia-biotec.com/uploads/2019/07/Milenia_HybriDetect_Citation_List_16_07_2020.pdf

Human research participants

Policy information about studies involving human research participants

Population characteristics	Human samples were obtained commercially from Bocabiolistics under their ethical approvals. All protocols subsequently performed using Bocabiolistics samplers by the researchers were approved as a Not Human Subjects Research. Saliva samples were obtained from volunteers as approved by the Harvard Medical School Institutional Review Board (IRB20-0581). Informed written consents were obtained by volunteers.
Recruitment	*Describe how participants were recruited. Outline any potential self-selection bias or other biases that may be present and how these are likely to impact results.*
Ethics oversight	Harvard Medical School Institutional Review Board

Note that full information on the approval of the study protocol must also be provided in the manuscript.
